# Estradiol mediates the association of waist-to-hip ratio, but not body fat percentage, with menopause-related symptoms in Chinese women aged 40–60 years

**DOI:** 10.3389/fendo.2026.1960475

**Published:** 2026-09-21

**Authors:** Xiaoyi Wang, Dongjian Yang, Jiahui Li, Yudong Wang, Furui Jin

**Affiliations:** 1 International Peace Maternity and Child Health Hospital, School of Medicine, Shanghai Jiao Tong University, Shanghai, China; 2 Shanghai Key Laboratory of Embryo Original Diseases, Shanghai, China

**Keywords:** adiposity, body fat percentage, estradiol, menopausal symptoms, waist-to-hip ratio

## Abstract

**Background:**

Menopause-related symptoms adversely affect quality of life. Although both overall and central adiposity have been associated with menopausal symptoms, the role of estradiol in these associations remains unclear.

**Methods:**

This single-center cross-sectional study included 600 women aged 40–60 years who attended the International Peace Maternity and Child Health Hospital, Shanghai, China. Participants were classified into premenopausal and postmenopausal groups according to the Stages of Reproductive Aging Workshop +10 (STRAW + 10) criteria. Menopause-related symptoms were assessed using the modified Kupperman Index (KMI). Body fat percentage (PBF) and waist-to-hip ratio (WHR) were measured using a bioelectrical impedance analyzer, and serum reproductive hormone levels, including estradiol (E_2_), were determined by chemiluminescent immunoassay. Multivariable linear regression models were used to examine the association between body composition parameters and KMI scores. Mediation analyses were performed to evaluate the mediating role of E_2_, and stratified analyses by menopausal status were conducted to assess the consistency of these associations.

**Results:**

After adjustment for potential confounders, higher WHR was independently associated with greater menopause-related symptom severity (β = 12.058, 95% CI: 1.816 to 22.300, *P* = 0.021), and higher PBF showed a similar association (β = 0.103, 95% CI: 0.011 to 0.196, *P* = 0.029). Mediation analyses revealed that E_2_ significantly mediated the association between WHR and KMI scores (indirect effect = 2.202, 95% CI: 0.520 to 4.323, *P* = 0.011; mediation proportion = 17.8%), whereas no significant mediating effect was observed for PBF (indirect effect = 0.008, 95% CI: −0.007 to 0.025, *P* = 0.296). Stratified analyses suggested that these associations were more evident among premenopausal women, although no significant interaction by menopausal status was observed.

**Conclusion:**

Both overall and central adiposity were associated with more severe menopause-related symptoms; however, estradiol mediated only the association between central adiposity and symptom severity. These findings suggest that different adiposity patterns may be associated with menopausal symptoms through distinct biological pathways.

## Introduction

With the extension of life expectancy, it has been estimated that nearly one-third of a woman’s life occurs after menopause (1, 2). During the menopausal transition, progressive decline in ovarian function is accompanied by marked fluctuations and an overall decline in estrogen levels, resulting in a range of symptoms, including menstrual irregularities, vasomotor symptoms, sleep disturbances, and mood changes (3, 4). More than 85% of women experience menopausal symptoms of varying severity during this period (5), which substantially impairs quality of life (6, 7). Beyond immediate impact, menopausal symptoms are associated with a greater long-term risk of chronic diseases, notably cardiovascular disorders, osteoporosis, and cognitive impairment (8, 9).

During the menopausal transition, declining estrogen levels promote fat accumulation and redistribution toward a more central adiposity pattern (10, 11). As an active endocrine organ, adipose tissue may influence menopausal symptoms through multiple biological pathways, including chronic inflammation, adipokine secretion, thermoregulation, and steroid hormone metabolism (12, 13). Importantly, the biological characteristics of adipose tissue vary according to its anatomical distribution. Visceral adipose tissue is generally characterized by greater metabolic activity and secretion of pro-inflammatory adipokines and cytokines, whereas subcutaneous adipose tissue has distinct adipokine profiles and may play a different role in lipid and steroid metabolism (14, 15). These depot-specific endocrine and metabolic properties suggest that the relationship between adiposity and menopausal symptoms may depend not only on the overall amount of body fat but also on its distribution. Consistent with this concept, previous studies have reported positive associations of both PBF, an indicator of overall adiposity, and WHR, an indicator of central adiposity, with menopausal symptom severity (16–19). Although the individual associations of estrogen decline, adiposity, and menopausal symptoms have been described, it remains unclear whether overall and central adiposity are associated with symptom severity through the same hormonal pathway. In particular, whether E_2_ differentially mediates the associations of PBF and WHR with menopausal symptoms has not been well established.

E_2_, the predominant circulating estrogen in women, is considered an important hormonal determinant of menopause-related symptoms. Declining E_2_ levels during the menopausal transition have been consistently associated with vasomotor symptoms, sleep disturbances, and mood changes (20–22). In addition to ovarian production, adipose tissue contributes to peripheral estrogen metabolism through aromatase-mediated conversion of androgens to estrone, which can subsequently be converted to estradiol (23). Given the distinct endocrine characteristics of different adipose depots, differences in adiposity distribution may therefore influence E_2_-related pathways linking adiposity to menopausal symptoms. However, whether E_2_ mediates the associations of different adiposity measures with menopausal symptom severity remains unclear.

Although mediation analysis can identify potential indirect associations, cross-sectional data cannot establish temporal ordering or causal mediation. Therefore, we hypothesized that higher PBF and WHR would be positively associated with greater menopause-related symptom severity and that E_2_ would statistically mediate these associations, with potentially different indirect effects between the two adiposity measures. Accordingly, this study compared overall adiposity (PBF) and central adiposity (WHR) within the same population to determine whether their associations with menopausal symptom severity differed with respect to E_2_-related pathways among Chinese women aged 40–60 years.

## Methods

### Study population

The present study employed a retrospective cross-sectional design and was carried out at the International Peace Maternity and Child Health Hospital (IPMCH) between July 2025 and April 2026. A total of 600 perimenopausal and postmenopausal women were included, see **Figure 1**.

**Figure 1:**
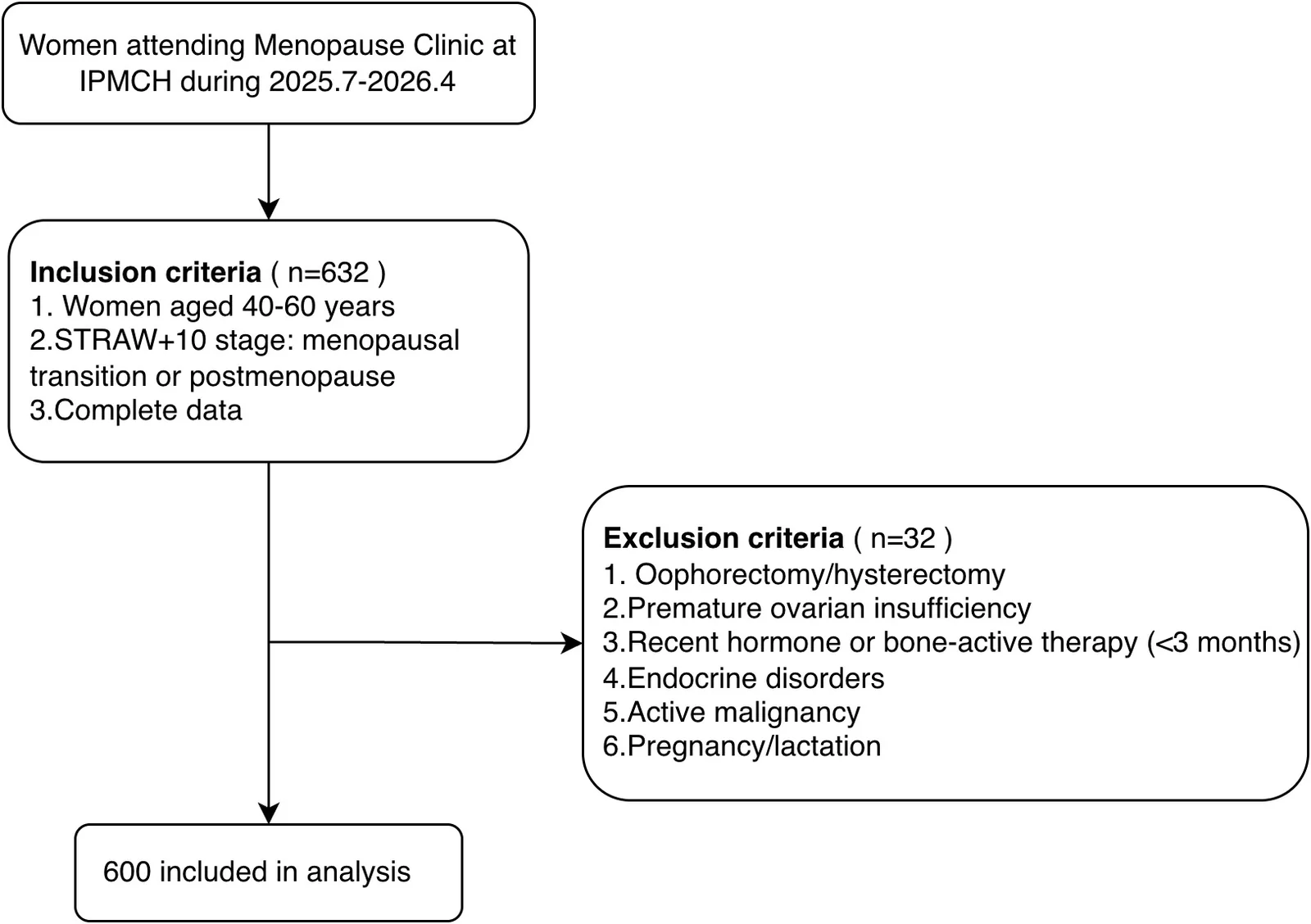
Study flowchart.

The inclusion criteria were as follows: (1) women aged 40–60 years; (2) classified as being in the menopausal transition or postmenopausal stage according to the STRAW + 10 criteria (24); (3) availability of complete clinical data, including menopause-related symptom assessment, hormone measurements, body composition analysis, and bone mineral density (BMD) examination. The menopausal transition was defined as a persistent change in menstrual cycle length of ≥7 days over 10 consecutive menstrual cycles, whereas postmenopause was defined as amenorrhea for ≥12 months. The exclusion criteria were: (1) a history of bilateral oophorectomy or hysterectomy; (2) primary ovarian insufficiency; and (3) use of medications affecting hormone levels or treating menopause-related symptoms within the previous 3 months; (4) use of medications affecting bone metabolism within the previous 3 months, including sex hormones, glucocorticoids, thyroid hormone replacement therapy, bisphosphonates, or selective estrogen receptor modulators; (5) thyroid dysfunction, hyperparathyroidism, Cushing syndrome, or malabsorption syndrome; (6) malignancy undergoing active treatment; and (7) pregnancy or lactation.

Because this was a retrospective cross-sectional study based on routinely collected clinical data, all eligible women meeting the predefined inclusion and exclusion criteria during the study period were included, rather than recruiting participants on the basis of a prospective sample size calculation. Therefore, an *a priori* sample size calculation was not performed. Statistical precision was evaluated based on the 95% confidence intervals of the estimates. The smaller postmenopausal subgroup (n = 180) may have limited the precision of the stratified regression estimates. The mediation analyses were conducted in the overall sample, and their precision was evaluated using bootstrap-derived 95% confidence intervals. Accordingly, the stratified and mediation findings were interpreted with appropriate caution.

The research protocol received ethical approval from the Institutional Review Board of IPMCH (Approval No. GKLW-A-2026-054-01). Given the retrospective nature of the study and the use of de-identified routinely collected clinical data, the Institutional Review Board approved a waiver of informed consent. The study involved no additional intervention, examination, or contact with participants, and only de-identified data necessary for the research objectives were accessed. Because participants were not prospectively recruited or contacted for this retrospective study, individual notification and a study-specific opt-out procedure were not required under the IRB-approved waiver of informed consent. This study was reported in accordance with the Strengthening the Reporting of Observational Studies in Epidemiology (STROBE) guidelines. The study was registered with the Chinese Clinical Trial Registry (ChiCTR2600128925).

### Data sources and covariates

Clinical and demographic data were extracted by trained researchers using a standardized data collection form from the Hospital Information System and the menopause patient registry. Variables included age, height, weight, menstrual status, marital status, reproductive history, educational level, occupation type, and medical history. According to the STRAW + 10 staging system, participants were classified into premenopausal and postmenopausal groups. Educational level was categorized as junior high school or below, bachelor’s degree, and postgraduate degree or above. Occupation type was categorized into three groups: home-based, mental labor, and light physical labor or physical labor.

### Outcomes

The severity of menopausal symptoms was assessed using the KMI (25). The questionnaire was administered by the same physician during the initial outpatient consultation through a standardized interview, with each item assessed by direct questioning and the responses recorded by the physician. The KMI evaluates symptom severity by multiplying a symptom-specific weighting factor (1, 2, or 4 points, depending on the symptom) by a 4-point severity score (0 = absent, 1 = occasional, 2 = frequent, 3 = severe). The weighting scheme assigns the highest score (4 points) to hot flashes and sweating, 2 points to paresthesia, insomnia, depression/suspicion, sexual symptoms, mood swings, and urinary symptoms, and 1 point to dizziness, arthralgia, fatigue, palpitations, headache, and formication. The total KMI score was calculated as the sum of the product of the base score and the severity score for each symptom. Based on the total score, symptom severity was categorized as normal (≤ 6), mild (7–15), moderate (16–30), or severe (> 30). The modified KMI has been used in clinical and research settings to assess the severity of menopausal symptoms, including in studies of Chinese peri- and postmenopausal women (26, 27). In the present study, the KMI total score was used as a composite indicator of overall menopausal symptom severity rather than as an instrument for comparing the relative burden of individual symptom domains.

### Measurement of body composition, bone mineral density, and hormones

Body composition parameters, including body weight, PBF, WHR, visceral fat grade (VFG), and muscle mass, were extracted from medical records. These measurements were obtained during routine clinical visits using an InBody 270 body composition analyzer (InBody Co., Seoul, South Korea) based on bioelectrical impedance analysis, following standardized operating procedures. To minimize measurement variability, participants were instructed to undergo BIA after voiding and before food intake whenever feasible. They were also instructed to avoid vigorous exercise and excessive fluid intake before the examination and to remove shoes and heavy clothing before measurement. Measurements were performed according to the manufacturer’s standardized procedures by trained staff. Bone mineral density (BMD) was measured using a Lunar Prodigy DF-570789 dual-energy X-ray absorptiometry (DXA) system (GE Healthcare Medical Systems and Basic Medical Diagnostics Co., Ltd.) at the lumbar spine (L1-L4) and right hip, according to standardized procedures.

Serum sex hormone data, including FSH and E_2_, were extracted from medical records. Hormone concentrations were determined by a chemiluminescent immunoassay system (Beckman Coulter DXI800, USA). For postmenopausal women, blood samples were collected irrespective of menstrual cycle timing. For premenopausal women, hormone measurements were routinely performed during the early follicular phase (days 2–5 of the menstrual cycle) according to standard clinical practice. For women with amenorrhea lasting more than 40 days, blood samples were collected after pregnancy had been excluded.

### Statistical analysis

Statistical analyses were performed using SPSS version 24.0 (SPSS Inc., Chicago, IL, USA). Continuous data were summarized as mean ± standard deviation (SD) or median (interquartile range, IQR), depending on their distribution, while categorical data were expressed as counts (percentages). For comparisons between the two groups, the independent samples t-test was used for normally distributed continuous measures, the Mann–Whitney U test for skewed continuous or ordinal variables, and the chi-square test for categorical variables. Multiple linear regression analysis was performed to examine the association between body composition parameters and KMI scores. Regression assumptions were assessed using variance inflation factors (VIFs) for multicollinearity, residual histograms and normal P–P plots for normality, and standardized residual-versus-predicted plots for linearity and homoscedasticity. Cook’s distance was used to identify potentially influential observations.

Before analysis, serum E_2_ concentrations below the limit of detection (LOD) were imputed using LOD/√2. To investigate whether E_2_ mediated the associations between adiposity indicators and menopausal symptoms, mediation analyses were performed using the mediation package in R (version 4.5.0). E_2_ was considered as a potential mediator. Bootstrap resampling (5,000 iterations) was applied to estimate the mediation effects, from which 95% confidence intervals (CIs) were derived. Given the close relationship between menopausal status and E_2_ levels, menopausal status was not included as a covariate in the mediation model because adjustment for menopausal status could remove part of the variation in E_2_ that is biologically related to reproductive aging and thereby lead to potential overadjustment. This choice represents a trade-off between potential overadjustment and residual confounding. Therefore, residual confounding related to menopausal status cannot be completely excluded.

## Results

The medical records of 632 women with menopausal syndrome who attended the menopause clinic at the International Peace Maternity and Child Health Hospital between July 2025 and April 2026 were retrospectively reviewed. After excluding 32 participants according to the predefined exclusion criteria, a total of 600 women were included in the final analysis, which included 420 premenopausal and 180 postmenopausal women.

As shown in **Table 1**, postmenopausal women were significantly older and exhibited lower skeletal muscle index, lower lumbar spine T-scores, and higher KMI scores than premenopausal women (all *P* < 0.05). No significant differences were observed in body weight, BMI, WHR, PBF, VFG between the two groups. As expected, postmenopausal women had significantly higher FSH levels and lower E_2_ levels than premenopausal women (both *P* < 0.001). Education level and the prevalence of hyperlipidemia differed significantly between groups (*P* = 0.004 and 0.009, respectively), whereas other demographic and clinical characteristics, including occupation type, marital status, reproductive history, and hypertension prevalence, were similar.

**Table 1:** Baseline characteristics of the study population according to menopausal status.

Variable	All cases (n = 600)	Premenopausal women (n=420)	Postmenopausal women (n=180)	*P* value
Age (years)	49.86 ± 3.46	48.87 ± 2.99	52.16 ± 3.41	<0.001
Weight (kg)	58.41 ± 7.84	58.67 ± 7.87	57.81 ± 7.74	0.219
BMI (kg/m^2^)	22.56 ± 2.88	22.71 ± 2.92	22.21 ± 2.75	0.051
WHR	0.87 ± 0.05	0.87 ± 0.05	0.87 ± 0.05	0.143
PBF (%)	31.74 ± 5.52	31.81 ± 5.63	31.68 ± 5.44	0.792
VFG	8 (6,10)	8 (6,10)	8 (6,10)	0.936
Skeletal muscle index (kg/m²)	6.15 ± 0.59	6.21 ± 0.60	6.10 ± 0.56	0.032
Lumbar spine T-score	−0.20 ± 1.40	0.07 ± 1.35	−0.74 ± 1.36	<0.001
KMI	14.09 ± 6.55	13.14 ± 6.33	16.32 ± 6.51	<0.001
Education Level, n (%)				0.004
Middle school or below	122 (20.3)	71 (16.9)	51 (28.3)	
University	412 (68.7)	298 (71.0)	114 (63.3)	
Master's degree or above	66 (11.0)	51 (12.1)	15 (8.3)	
Occupation type, n (%)				0.351
Home based	35 (5.8)	28 (6.7)	7 (3.9)	
Mental labor	509 (84.8)	355 (84.5)	154 (85.6)	
Light physical labor/ physical labor	56 (9.3)	37 (8.8)	19 (10.6)	
Marital Status, n (%)				0.869
Married	528 (88.0)	369 (87.9)	159 (88.3)	
Single/Divorced/Widowed	72 (12.0)	51 (12.1)	21 (11.7)	
Number of Births, n (%)				0.114
0	52 (8.7)	36 (8.6)	16 (8.9)	
1	469 (78.2)	321 (76.4)	148 (82.2)	
≥2	79 (13.2)	63 (15.0)	16 (8.9)	
Number of Abortions, n (%)				0.596
0	169 (28.2)	119 (28.3)	50 (27.8)	
1	204 (34.0)	146 (34.8)	58 (32.2)	
≥2	227 (37.8)	155 (36.9)	72 (40.0)	
Hypertension, n (%)				0.237
Yes	54 (9.0)	34 (8.1)	20 (11.1)	
No	546 (91.0)	386 (91.9)	160 (88.9)	
Hyperlipidemia, n (%)				0.009
Yes	100 (16.7)	59 (14.0)	41 (22.8)	
No	500 (83.3)	361 (86.0)	139 (77.2)	
FSH (IU/L)	60.25 (26.28,89.18)	46.55 (17.25,79.45)	81.00 (63.75,105.03)	<0.001
E_2_ (pmol/L)	40.0 (17.7,158.3)	52.7 (17.7,174.3)	17.7(17.7,26.7)	<0.001

PBF, percentage of body fat; WHR, waist-to-hip ratio; VFG, visceral fat grade.

In the model additionally adjusted for FSH and E_2_, WHR (β = 12.058, 95% CI: 1.816 to 22.300, *P* = 0.021), body fat percentage (β = 0.103, 95% CI: 0.011 to 0.196, *P* = 0.029), and visceral fat grade (β = 0.169, 95% CI: 0.004 to 0.333, *P* = 0.044) were positively associated with KMI scores. WHR remained significantly associated with KMI scores across all models, whereas associations of body fat percentage and visceral fat grade reached statistical significance after adjustment for sex hormones. No significant associations were observed for BMI, skeletal muscle index, or lumbar spine T-score, see **Table 2**.

**Table 2:** Multiple linear regression analyses of the associations between body composition parameters and KMI scores.

Exposure	Model	β (95% CI)	*P* value
PBF	Unadjusted	0.088 (−0.006, 0.182)	0.067
	Model 1	0.087 (−0.007, 0.180)	0.070
	Model 2	0.103 (0.011, 0.196)	0.029
WHR	Unadjusted	12.571 (2.335, 22.807)	0.016
	Model 1	11.168 (0.873, 21.463)	0.034
	Model 2	12.058 (1.816, 22.300)	0.021
BMI	Unadjusted	−0.006 (−0.189, 0.177)	0.951
	Model 1	0.039 (−0.143, 0.222)	0.672
	Model 2	0.131 (−0.057, 0.319)	0.171
VFG	Unadjusted	0.134 (−0.032, 0.301)	0.113
	Model 1	0.127 (−0.038, 0.292)	0.130
	Model 2	0.169 (0.004, 0.333)	0.044
Skeletal muscle index	Unadjusted	−0.663 (−1.523, 0.258)	0.163
	Model 1	−0.364 (−1.247, 0.518)	0.418
	Model 2	0.034 (−0.877, 0.945)	0.942
Lumbar spine T-score	Unadjusted	−0.091 (−0.563, 0.381)	0.704
	Model 1	0.212 (−0.280, 0.704)	0.398
	Model 2	0.339 (−0.149, 0.827)	0.173

PBF, percentage of body fat; WHR, waist-to-hip ratio; VFG, visceral fat grade.

Model 1: Adjusted for age, education level, occupation type, marital status, parity, number of abortions, and menopausal stage.

Model 2: Model 1 additionally adjusted for FSH and E_2_.

Regression diagnostic analyses for the overall models showed no evidence of substantial multicollinearity, with VIF values ranging from 1.023 to 2.947. The VIFs for FSH and E_2_ ranged from 1.618 to 1.751 and from 1.301 to 1.333, respectively. Visual inspection of standardized residual histograms and normal P–P plots showed no substantial deviation from normality, while scatterplots of standardized residuals against standardized predicted values showed no evident systematic or funnel-shaped pattern, supporting the assumptions of linearity and homoscedasticity. The maximum Cook’s distance ranged from 0.023 to 0.031, indicating no influential observations.

As shown in **Table 3** and **Figure 2**, after adjusting for age, educational level, occupation type, marital status, parity, and number of abortions, the indirect effect of PBF on KMI through E_2_ was not statistically significant (ACME = 0.008, 95% CI: −0.007 to 0.025, *P* = 0.296), suggesting that E_2_ did not significantly mediate the association between PBF and KMI. In contrast, E_2_ significantly mediated the association between WHR and KMI (ACME = 2.202, 95% CI: 0.520 to 4.323, *P* = 0.011), accounting for 17.8% of the total effect (P = 0.032).

**Figure 2:**
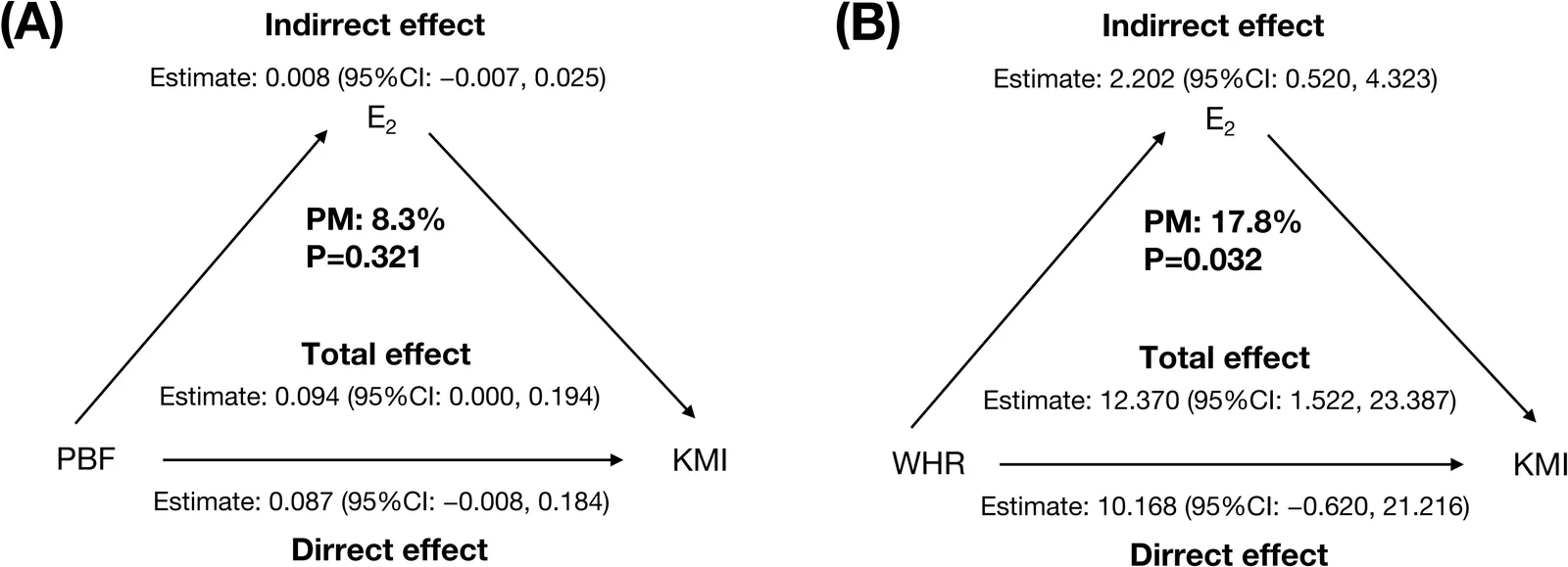
Mediating role of E_2_ in the associations of PBF **(A)** and WHR **(B)** with KMI scores.

**Table 3:** Mediation analyses of E_2_ in the associations of PBF and WHR with KMI scores.

Exposure	Mediator	Effect	Estimate (95%CI)	P value
PBF	E_2_	ACME (Indirect effect)	0.008 (−0.007, 0.025)	0.296
		ADE (Direct effect)	0.087 (−0.008, 0.184)	0.072
		Total effect	0.094 (0.000, 0.194)	0.050
		Proportion mediated (%)	8.3 (−17.3, 58.0)	0.321
WHR	E_2_	ACME (Indirect effect)	2.202 (0.520, 4.323)	0.011
		ADE (Direct effect)	10.168 (−0.620, 21.216)	0.068
		Total effect	12.370 (1.522, 23.387)	0.023
		Proportion mediated (%)	17.8 (2.9, 94.2)	0.032

PBF, percentage of body fat; WHR, waist-to-hip ratio.

Mediation analysis was adjusted for age, education level, occupation type, marital status, parity, number of abortions.

In stratified analyses by menopausal status, WHR was significantly and positively associated with KMI scores among premenopausal women across all three models (Model 2: β = 13.392, 95% CI: 1.310 to 25.473, *P* = 0.030). The association between PBF and KMI scores became significant after additional adjustment for FSH and E_2_ (β = 0.110, 95% CI: 0.002 to 0.218, *P* = 0.046). In contrast, no significant associations were observed between PBF, WHR, or VFG and KMI scores among postmenopausal women (all *P* > 0.05), see **Table 4**. Although formal tests for interaction did not reach statistical significance, these findings suggest that adiposity-related associations with menopause-related symptoms may be more evident among premenopausal women.

**Table 4:** Stratified analysis of the association of PBF, WHR, and VFG with KMI scores by menopausal status, with P for interaction.

Regression model	Premenopausal women		Postmenopausal women		*P* for interaction
	β (95% CI)	*P* Value	β (95% CI)	*P* value	
PBF					0.890
Unadjusted	0.104 (−0.004, 0.211)	0.059	0.058 (−0.119, 0.235)	0.518	
Model 1	0.102 (−0.009, 0.212)	0.071	0.062 (−0.144, 0.229)	0.651	
Model 2	0.110 (0.002, 0.218)	0.046	0.096 (−0.114, 0.260)	0.444	
WHR					0.761
Unadjusted	13.072 (1.084, 25.060)	0.033	6.168 (−12.208, 24.544)	0.509	
Model 1	13.557 (1.287, 25.826)	0.030	3.271 (−16.884, 23.425)	0.749	
Model 2	13.392 (1.310, 25.473)	0.030	6.174 (−14.292, 26.640)	0.552	
VFG					0.957
Unadjusted	0.153 (−0.039, 0.344)	0.117	0.088 (−0.220, 0.396)	0.573	
Model 1	0.147 (−0.047, 0.342)	0.137	0.057 (−0.270, 0.384)	0.730	
Model 2	0.168 (−0.023, 0.360)	0.085	0.143 (−0.191, 0.478)	0.399	

PBF, percentage of body fat; WHR, waist-to-hip ratio; VFG, visceral fat grade.

Model 1: Adjusted for age, education level, occupation type, marital status, parity, number of abortions.

Model 2: Model 1 additionally adjusted for FSH and E_2_.

Similarly, no substantial multicollinearity was observed in the menopausal status stratified models. VIF values ranged from 1.035 to 2.324 in the premenopausal group and from 1.078 to 4.071 in the postmenopausal group. The VIFs for FSH and E_2_ remained low across all stratified models, ranging from 1.215 to 1.367 and from 1.184 to 1.290, respectively.

As shown in **Supplementary Table 1**, hot flashes and sweating, insomnia, and joint pain showed relatively high proportions of moderate-to-severe symptoms, whereas most other individual symptoms were predominantly absent or mild.

## Discussion

This study investigated the associations of different adiposity patterns with menopausal symptoms and further explored the potential mediating role of estradiol. We found that overall adiposity, represented by PBF and central adiposity, represented by WHR, were positively associated with more severe menopausal symptoms, whereas BMI, skeletal muscle index, and lumbar spine bone status were not. Importantly, estradiol significantly mediated the association between WHR and menopausal symptoms, but not the association between PBF and symptoms, suggesting that different adiposity patterns may be associated with menopausal symptom severity through distinct hormonal pathways. Stratified analyses suggested that these associations were more evident among premenopausal women, although formal interaction analyses did not demonstrate significant effect modification by menopausal status.

The distribution of individual KMI symptoms further showed that moderate-to-severe hot flashes and sweating, insomnia, and joint pain were relatively common in our study population, indicating that the overall menopausal symptom burden reflected contributions from several clinically relevant symptoms. Previous studies have consistently demonstrated associations between adiposity and menopausal symptoms. Consistent with these findings, we observed that adiposity indicators, particularly PBF and WHR, were independently associated with more severe menopausal symptoms. Previous analyses from the SWAN cohort similarly showed that higher PBF was associated with an increased risk of vasomotor symptoms independent of FSH and E_2_ levels (16, 28), supporting a potential thermoregulatory mechanism. Consistent with this finding, the nonsignificant PBF–E_2_ mediation pathway observed in our study suggests that overall adiposity may be associated with menopausal symptoms through E_2_-independent mechanisms. Moore et al. also reported that PBF explained a substantial proportion of the variation in menopausal symptoms among perimenopausal women (17). More recently, Tang et al. and Moradpour et al. further highlighted the importance of central adiposity, demonstrating significant associations between abdominal obesity indicators and menopausal symptoms (18, 19). Collectively, these findings support the contribution of both overall adiposity and central fat distribution to menopausal symptom severity.

Although the associations of PBF and WHR with KMI scores were statistically significant, their absolute effect sizes were modest. A 10-percentage-point increase in PBF corresponded to an approximately 1.03-point increase in KMI score, while a 0.1-unit increase in WHR corresponded to an approximately 1.21-point increase. Given that KMI scores ranged from 0 to 35 in our study population and that the KMI is categorized as normal (≤6), mild (7–15), moderate (16–30), and severe (>30), these estimated changes were relatively small in relation to the overall symptom scale. Therefore, the observed associations should be interpreted as modest population-level differences in symptom severity rather than large clinically meaningful changes at the individual level.

Regarding the biological mechanisms underlying these associations, adipose tissue may be associated with menopausal symptoms through multiple endocrine, inflammatory, and metabolic pathways. Beyond serving as an energy storage organ, adipose tissue acts as a dynamic endocrine organ, producing adipokines and pro-inflammatory mediators (29, 30), promotes chronic low-grade inflammation (31), and affects metabolic homeostasis (22, 32). These alterations may contribute to menopausal symptom development by influencing systemic inflammation, vascular function, and thermoregulatory regulation. In addition, adipose tissue is involved in sex steroid metabolism through the expression of aromatase and other steroidogenic enzymes (33, 34), suggesting a potential link between adiposity, estrogen availability, and menopause-related symptoms. Furthermore, the thermoregulatory model suggests that excess adiposity may impair heat dissipation and narrow the thermoneutral zone, thereby increasing susceptibility to vasomotor symptoms (16, 35). Together, these mechanisms provide biological plausibility for the observed associations between adiposity and menopausal symptom severity and suggest that hormonal pathways may contribute to these relationships.

A central and novel finding of the present study was that estradiol showed a differential mediating role in the associations between adiposity patterns and menopausal symptoms. Specifically, E_2_ significantly mediated the association between WHR and KMI scores, whereas no significant mediation effect was observed for PBF. This finding suggests that the differential associations of adiposity may depend not only on the amount of fat accumulation but also on its distribution and endocrine characteristics. During the reproductive years and menopausal transition, circulating estradiol is predominantly produced by the ovaries. However, adipose tissue also participates in estrogen metabolism through the expression of aromatase and other steroidogenic enzymes (33, 36). Aromatase converts androgens into estrone, which can subsequently be converted into biologically active E_2_ through 17β-hydroxysteroid dehydrogenase activity (34, 37).

Importantly, adipose tissue is not a homogeneous organ, and different fat depots exhibit distinct metabolic and endocrine characteristics (38, 39). Compared with subcutaneous adipose tissue, abdominal and visceral adipose tissue are characterized by greater metabolic activity, enhanced lipolysis, increased inflammatory potential, and altered steroid hormone metabolism (40). Therefore, WHR, which reflects abdominal fat distribution, may better capture the endocrine activity of metabolically active adipose tissue than PBF, which primarily reflects overall fat quantity. This difference may partly explain why E_2_ mediated the WHR–KMI association but not the PBF–KMI association. In contrast, overall adiposity may influence menopausal symptoms predominantly through E_2_-independent pathways, including chronic inflammation, adipokine dysregulation, metabolic disturbances, and impaired thermoregulation. Collectively, these findings suggest that the relationship between adiposity and menopausal symptoms is influenced by both the quantity and distribution of adipose tissue, with central adiposity potentially showing additional associations involving estrogen-related pathways.

Stratified analyses suggested that the associations of PBF and WHR with menopausal symptoms were more evident among premenopausal women, although formal interaction analyses did not demonstrate significant effect modification by menopausal status. This finding is consistent with previous cohort studies showing that obesity-related menopausal symptoms, particularly vasomotor symptoms are more pronounced during the menopausal transition (17, 41). The dynamic endocrine environment during this period, characterized by fluctuating ovarian hormone levels (42), may increase susceptibility to the effects of metabolic alterations on symptom development (43, 44). As ovarian function declines after menopause, the relative contribution of adiposity-related metabolic factors to symptom severity may become less apparent due to the increasing influence of age-related physiological changes and psychosocial factors.

The strengths of this study lie in several aspects. First, rather than relying solely on conventional anthropometric measures such as body mass index, we comprehensively evaluated body composition using multiple parameters, including overall adiposity (PBF), central adiposity (WHR), visceral fat grade, skeletal muscle index, and bone mineral density. This approach allowed us to explore the distinct associations of different adiposity patterns with menopausal symptoms. Second, to our knowledge, this study provides novel evidence that estradiol may represent a differential biological pathway linking specific adiposity patterns to menopausal symptoms. Specifically, E_2_ partially mediated the association between WHR and symptom severity but not the association between PBF and symptoms. These findings provide novel insights into the potential hormonal pathways linking central adiposity, estrogen metabolism, and menopausal symptom development. Third, the integration of mediation analysis with stratified analyses and adjustment for multiple potential confounders strengthened the robustness of our findings and provided further insight into potential biological mechanisms underlying these associations.

However, several limitations should be acknowledged. First, the cross-sectional design precludes establishing temporal ordering or causal mediation; therefore, the mediation results should be interpreted as statistical indirect associations. Because menopausal status was not included in the mediation models, residual confounding related to menopausal status cannot be excluded. Second, recruitment from a single menopause clinic may limit generalizability and introduce selection bias. Third, body composition parameters were assessed using the InBody 270, which may slightly overestimate body fat percentage and underestimate fat-free mass compared with DXA. Fourth, no adjustment for multiple comparisons was performed, and associations with P values close to the significance threshold should therefore be interpreted cautiously. E_2_ values below the limit of detection were imputed using LOD/√2, which may have influenced the estimates, therefore, the E_2_-related mediation findings should be interpreted with caution. A single measurement of serum E_2_ may not fully reflect long-term estrogen exposure because of physiological fluctuations during the menopausal transition. Finally, the psychometric properties of the modified KMI were not re-evaluated in this population, and its historical weighting system may not fully reflect the clinical importance of different symptoms. Although multiple potential confounders were adjusted for, residual confounding from unmeasured factors, including dietary habits, physical activity, sleep quality, and psychological status, cannot be completely excluded.

Despite these limitations, our findings suggest that adiposity distribution and endocrine characteristics may be relevant to menopausal symptom severity. Clinically, simple assessment of central adiposity, such as WHR, may provide additional information beyond BMI when evaluating menopausal symptom burden. From a research perspective, prospective studies with repeated hormone measurements and more precise assessment of fat distribution are needed to clarify the temporal and biological pathways linking adiposity to menopausal symptoms.

## Conclusions

Higher PBF and WHR were independently associated with more severe menopausal symptoms, with these associations being more evident among premenopausal women. Estradiol partially mediated the association between WHR, but not PBF, and symptom severity, suggesting that different adiposity patterns may be associated with menopausal symptoms through distinct biological pathways. These findings highlight the importance of assessing adipose distribution and estrogen-related mechanisms in women with menopausal symptoms and warrant further prospective studies to clarify the underlying pathways.

## Data Availability

The raw data supporting the conclusions of this article will be made available by the authors, without undue reservation.

## Supplementary material

Supplementary Table 1 Distribution of individual symptoms assessed by the modified Kupperman Index. Individual symptom severity was scored from 0 (absent) to 3 (severe). Data are presented as n (%) or median (interquartile range).
