# Supplementary material for: Estradiol mediates the association of waist-to-hip ratio, but not body fat percentage, with menopause-related symptoms in Chinese women aged 40–60 years
Source: Front Endocrinol (Lausanne). 2026 Sep 21;17:1960475. doi: 10.3389/fendo.2026.1960475 (PMC13634826; doi:10.3389/fendo.2026.1960475)
Supplement: Supplementary file 1 [file Table1.docx]

**Supplementary Table 1. Distribution of individual symptoms assessed by the modified Kupperman Index**

| Individual symptom | Absent, n (%) | Mild, n (%) | Moderate, n (%) | Severe, n (%) | Median (IQR) |
| --- | --- | --- | --- | --- | --- |
| Hot flashes and sweating | 186 (31.0) | 197 (32.8) | 178 (29.7) | 39 (6.5) | 1 (0–2) |
| Insomnia | 225 (37.5) | 198 (33.0) | 147 (24.5) | 30 (5.0) | 1 (0–2) |
| Emotional fluctuations | 268 (44.7) | 229 (38.2) | 95 (15.8) | 8 (1.3) | 1 (0–1) |
| Depression/suspicion | 431 (71.8) | 129 (21.5) | 38 (6.3) | 2 (0.3) | 0 (0–1) |
| Joint pain | 238 (39.7) | 181 (30.2) | 180 (30.0) | 1 (0.2) | 1 (0–2) |
| Paresthesia | 474 (79.0) | 88 (14.7) | 38 (6.3) | 0 (0.0) | 0 (0–0) |
| Dizziness | 364 (60.7) | 196 (32.7) | 33 (5.5) | 7 (1.2) | 0 (0–1) |
| Fatigue | 189 (31.5) | 410 (68.3) | 1 (0.2) | 0 (0.0) | 1 (0–1) |
| Headache | 364 (60.7) | 181 (30.2) | 55 (9.2) | 0 (0.0) | 0 (0–1) |
| Formication | 540 (90.0) | 54 (9.0) | 6 (1.0) | 0 (0.0) | 0 (0–0) |
| Palpitations | 276 (46.0) | 262 (43.7) | 59 (9.8) | 3 (0.5) | 1 (0–1) |
| Sexual symptoms | 176 (29.3) | 345 (57.5) | 78 (13.0) | 1 (0.2) | 1 (0–1) |
| Urinary tract symptoms | 516 (86.0) | 57 (9.5) | 17 (2.8) | 10 (1.7) | 0 (0–0) |

Note: Individual symptom severity was scored from 0 (absent) to 3 (severe). Data are presented as n (%) or median (interquartile range).
